# Sharing Experience with Anaplastic Lymphoma Kinase Tyrosine Kinase Inhibitors in Lung Cancer: An Italian Expert Panel Discussion

**DOI:** 10.3390/curroncol30110729

**Published:** 2023-11-20

**Authors:** Cesare Gridelli, Marcello Tiseo, Diego Luigi Cortinovis, Maria Rita Migliorino, Vito Barbieri, Paolo Bironzo, Alessandra Bearz, Ilaria Attili, Filippo de Marinis

**Affiliations:** 1 Division of Medical Oncology, “S.G. Moscati” Hospital, 83100 Avellino, Italy; cgridelli@libero.it; 2 Department of Medicine and Surgery, University of Parma, 43126 Parma, Italy; 3 Medical Oncology Unit, University Hospital of Parma, 43126 Parma, Italy; 4 SC Medical Oncology, Fondazione IRCCS San Gerardo dei Tintori, 20900 Monza, Italy; 5 Department of Oncology, San Camillo-Forlanini Hospital, 00152 Rome, Italy; 6 Medical Oncology Unit, AOU Renato Dulbecco, 88100 Catanzaro, Italy; 7 Department of Oncology, University of Turin, 10124 Turin, Italy; 8 Department of Medical Oncology, Centro di Riferimento Oncologico di Aviano (CRO), IRCCS, 33081 Aviano, Italy; 9 Division of Thoracic Oncology, European Institute of Oncology, IRCCS, 20141 Milan, Italy

**Keywords:** lung cancer, ALK, lorlatinib, brigatinib, alectinib

## Abstract

Background: ALK tyrosine kinase inhibitors (TKIs) have revolutionized the treatment and largely improved the survival outcomes of patients with NSCLC harboring *ALK* rearrangements. Different ALK TKI compounds have demonstrated antitumor activity in these patients and are available in clinical practice. However, clinical expertise across countries varies according to local regulatory approval of different drugs, identifying multiple treatment scenarios to comply with international guidelines and clinical practice. Methods: A virtual webinar was held on July 2023 to discuss the state of the art and future perspectives in the treatment of *ALK* rearrangement in advanced NSCLC in Italy. The faculty hosting the webinar was composed of eight medical oncologists from different regions of Italy with clinical expertise in treating patients with lung cancer. Live-shared notes were used to produce a report to serve as the basis of a review manuscript on the topic. Results: Alectinib and brigatinib are the preferred front-line treatment options in Italy, pending approval of the front-line medicine lorlatinib, which would be considered among the choices. Due to a local regulatory limitation of second-line lorlatinib, which is not allowed after front-line brigatinib, alectinib is commonly the preferred front-line choice to follow a sequence of alectinib, followed by lorlatinib, followed by platinum plus pemetrexed chemotherapy. Age and performance status were not considered per se as clinical features influencing treatment choice. However, treatment compliance is deemed a relevant factor in decision making with regard to the number of pills to be administered. In general, given the availability of alternative choices, the spectrum of patients’ comorbidities and polypharmacotherapy interactions should be taken into account in treatment selection according to the toxicity profile of each compound. In addition, several issues were debated with regard to improving treatment outcomes, including testing, brain metastases, and management of an oligoprogressive disease. Conclusions: The treatment scenario of ALK-positive disease is dynamically evolving. Furthermore, not all FDA- and EMA-approved compounds are approved in Italy with the same indications. This influences therapeutic opportunities and increases the need for greater clinical expertise to help and guide treatment selection.

## 1. Introduction

Anaplastic lymphoma kinase (*ALK*) gene rearrangements are identified in 3–7% of patients with advanced non-small-cell lung cancer (NSCLC) [1]. Most cases occur in non-squamous histology, whereas their occurrence in squamous histology is anecdotical [1].

After its identification in NSCLC in 2007 [2], it was rapidly identified as a potential therapeutic target, and several targeted agents within the class of tyrosine kinase inhibitor (TKI) drugs [3] were soon developed and led to dramatic changes in the natural history of this disease [4]. Indeed, nowadays, the prognosis of patients diagnosed with *ALK*-positive NSCLC treated with ALK-targeted TKIs is characterized by long-term survival (>60% overall survival rate at 5 years) [5,6].

Based on results from randomized phase 3 clinical trials, different ALK TKI compounds have been approved worldwide, with different timings and settings of approval across local regulatory systems [7]. In particular, the Food and Drug Administration (FDA) approved the first ALK TKI, crizotinib, in 2011. The year of registration and approval of the same compound by the European Medicine Agency (EMA) was 2012 for previously treated patients and 2015 for the front-line setting (Table 1). Second-generation ALK TKI alectinib received FDA approval in 2016 (2017 as a front-line treatment) and EMA registration in 2017. The respective approval years of front-line brigatinib and lorlatinib were 2020 and 2021 (Table 1). The different timings of drug approval necessarily conditioned the clinical use and expertise of these multiple compounds by medical oncologists in different countries. With respect to the Italian scenario, the first approval for crizotinib by Agenzia Italiana del Farmaco (AIFA) occurred in 2015, rapidly followed by the approval of ceritinib (2017), alectinib (2018), and brigatinib (2020). Lorlatinib was approved in Italy in 2021 only as a second-line option after front-line alectinib or after a sequence of crizotinib and another ALK TKI. A panel of Italian medical oncologists with expertise in treating patients with NSCLC gathered in a virtual webinar to share and discuss the available treatment options for *ALK*-positive advanced NSCLC in Italy, with the aim of providing a manuscript from their discussion that may help clinicians in current and future decision making.

## 2. Methods

A webinar was held virtually on 11th July 2023 to discuss the topic ‘Sharing experience: 5 year-clinical use of alectinib in Italy’. The scientific panel of the meeting—also referred to as the ‘faculty’, ‘panelists’, or ‘experts’ throughout the text—was made up of eight medical oncologists from different Italian regions with clinical expertise in treating patients with lung cancer.

During the first part of the meeting, the available evidence on ALK-targeted tyrosine kinase inhibitors was formally reviewed. Evidence was identified by performing a PubMed search, using the combinations of the following search terms: ‘non-small cell lung cancer’, ‘ALK’, ‘tyrosine kinase inhibitors’, and relevant synonyms. Only articles written in English were considered. The search has been updated with the proceedings of the main international meetings (American Society of Clinical Oncology, European Society of Medical Oncology, and the World Conference on Lung Cancer) from 2015 to 2023. Relevant references from selected articles were also included.

The second part of the meeting hosted a panelists’ discussion to reach shared considerations on the current and prospective use of ALK TKIs in Italy. For this purpose, five points of discussion, previously agreed upon by the scientific committee of the meeting, were proposed and fully addressed by the panelists: front-line options, patients’ selection criteria, sequence of treatment, safety profile, and improving strategies.

Due to the intended applicability of the discussion to the Italian scenario, the shared considerations were based on the current and prospective availability of the different ALK TKIs according to regulatory approvals in Italy.

During the webinar, live-shared minutes were collected to produce a summary report of the meeting, which was agreed upon by all panel members to serve as the basis to generate the current review manuscript.

## 3. Evidence on the Front-line Use of ALK TKIs in Patients with ALK-Positive NSCLC

After evidence of its efficacy in previously treated patients [8], crizotinib was the first ALK TKI to demonstrate efficacy in the front-line setting. In the phase 3 PROFILE-1014 trial, 343 patients with advanced *ALK*-positive non-squamous NSCLC, treatment-naïve, were randomized to receive crizotinib or platinum plus pemetrexed chemotherapy, with crossover allowed for patients assigned to the control arm [9]. The ORR was 74% versus 45% with crizotinib and chemotherapy, respectively. The median progression-free survival (PFS), the primary endpoint of the study, was 10.9 versus 7 months, respectively (HR 0.45, 95% CI 0.35–0.60) [9]. Long-term overall survival (OS), measured as the 4-year OS rate, was 56.6% with crizotinib and 49.1% for chemotherapy [10]. The crizotinib safety profile was acceptable, with the most common adverse events (AEs) being vision disorders, diarrhea, nausea, and edema [9].

Ceritinib was tested in the ASCEND trial series [11]. In the front-line setting, the phase 3 randomized ASCEND-4 trial of ceritinib versus chemotherapy also showed a significant benefit in PFS (median 16.6 vs. 8.1 months, HR 0.55, 95% CI 0.42–0.73) [12]. The most common AEs with ceritinib were diarrhea, nausea, vomiting, and an increase in alanine aminotransferase (85%, 69%, 66%, and 60%, respectively) [12], negatively impacting its use in clinical practice in the presence of valid alternatives.

The following tested ALK TKI compounds (Table 2)—namely, alectinib, brigatinib, ensartinib, and lorlatinib—after positive results in the post-crizotinib setting, were evaluated in front-line clinical trials with randomization versus the new standard crizotinib (Table 3).

The ALEX trial was a phase 3 study randomizing 303 *ALK*-positive patients with advanced NSCLC to receive front-line alectinib or crizotinib, with no crossover permitted [13]. Investigator-assessed PFS, the primary endpoint of the study, was 34.8 versus 10.9 months (HR 0.43, 95% CI 0.32–0.58), whereas the blinded independent central review (BICR) PFS was 25.7 versus 10.4 months (HR 0.50, 95% CI 0.36–0.70). OS results are still not mature, with an estimated 5-year OS rate of 62.5% with alectinib [5]. AEs related to alectinib were manageable, with the most common being anemia, myalgia, increase blood bilirubin, and increased weight (20%, 16%, 15%, and 10%, respectively) [13].

Brigatinib was tested in the front-line setting in the phase 3 ALTA-1L trial, with crossover permitted for patients assigned to the crizotinib arm. Median PFS, the primary endpoint of the study, was 24 versus 11.1 months with brigatinib and crizotinib, respectively (HR 0.48, 95% CI 0.35–0.66) [14]. The 4-year OS rate with brigatinib was 66% [6]. The most common AEs were gastrointestinal, increased blood creatine phosphokinase and aminotransferases, and cough [15]. Interstitial lung disease (ILD) was a relevant toxicity in the first studies; however, the strategy to start treatment at a lower, safer dosage before escalation (90 mg daily for the first week, then 180 mg daily) markedly reduced the risk of ILD (4.5%, only 3% of grade 3 or greater across studies) [16].

Another compound, ensartinib, was instead evaluated in the eXalt3 trial in the front-line setting in 290 naïve patients with *ALK*-positive NSCLC, with no crossover allowed. The median PFS was 25.8 months with ensartinib and 12.7 months with crizotinib (HR 0.51, 95% CI 0.35–0.72) [17]. The safety profile was represented by skin rash and transaminase increase as the most common AEs [17]. Of note, despite having similar results compared with those of the other second-generation ALK TKIs, this compound is, to date, only approved in China.

The third-generation ALK TKI lorlatinib, after relevant results in the setting of previous TKI resistance, was evaluated in the randomized phase 3 CROWN trial. In this study, 296 naïve patients with advanced *ALK*-positive NSCLC received lorlatinib or crizotinib, with no crossover allowed [18]. The updated results after a 3-year follow-up showed that median PFS was not reached with lorlatinib versus 9.1 months with crizotinib (HR, 0.19; 95% CI, 0.131–0.274) [19]. The safety profile of lorlatinib was characterized by hyperlipidemia, edema, increased weight, peripheral neuropathy, and cognitive effects. With regards to cognitive effects (20.8% overall), they were mostly grades 1 or 2, whereas dyslipidemia was not associated with a higher risk of cardiovascular events [19,20].

**Table 2:** Main pharmacology and pharmacokinetic properties of the principal ALK inhibitors.

	Generation	Targets	ALK K_i_ (nM) [21]
**Crizotinib**	First	ALK, c-Met, ROS1	1.6
**Ceritinib**	Second	ALK, IGF-1R, InsR, ROS1	0.10
**Alectinib**	Second	ALK, RET	0.09
**Brigatinib**	Second	ALK, EGFR	0.10
**Lorlatinib**	Third	ALK	0.14

ALK: anaplastic lymphoma kinase, c-Met: Hepatocyte growth factor receptor, ROS1: ROS proto-oncogene 1, IGF-1R: insulin-like growth factor 1 receptor, InsR: insulin receptor, RET: rearranged during transfection, EGFR: epidermal growth factor receptor.

**Table 3:** Main results from phase 3 trials with ALK inhibitors in the front-line setting.

Drug	Trial Name	Control Arm	Primary Endpoint	BIRC-mPFS (Months)
**Crizotinib**	PROFILE-1014 [9,10]	Pemetrexed-based chemotherapy	BIRC-mPFS	10.9
**Ceritinib**	ASCEND-4 [11,12]	Pemetrexed-based chemotherapy	BIRC-mPFS	16.6
**Alectinib**	ALEX [13]	Crizotinib	mPFS	25.7
**Brigatinib**	ALTA-1L [14]	Crizotinib	BIRC-mPFS	24.0
**Ensartinib**	EXALT-3 [17]	Crizotinib	BIRC-mPFS	25.8
**Lorlatinib**	CROWN [18,19,20]	Crizotinib	BIRC-mPFS	NR

BIRC: blinded independent radiologic review, mPFS: median progression-free survival, NR: not reached.

## 4. Sharing Experience Discussion

### 4.1. Front-Line Treatment in Patients with ALK-Positive NSCLC: From Guidelines to Clinical Practice

The first issue for discussion was related to the available treatments in Italy in the front-line setting of *ALK*-positive disease.

According to all faculty, among the approved ALK TKIs as first-line treatments in Italy (crizotinib, ceritinib, alectinib, and brigatinib), no role for crizotinib or ceritinib should be considered nowadays. Conversely, both alectinib and brigatinib should be considered as valid front-line treatment options.

However, since there are regulatory limitations by AIFA for the subsequent use of lorlatinib after ALK TKIs (allowed after failure of front-line alectinib but not after front-line brigatinib), the preferred first-line treatment choice across Italian regions is commonly alectinib. Due to this limitation, clinicians advocated and requested a specific regulatory reimbursement (named Law 648/1996) according to a different rule for the sequential use of lorlatinib after first-line brigatinib, for those patients who received brigatinib in the phase 3 randomized ALTA-1L trial in the front-line setting. However, the request has been denied by AIFA.

Besides the major selection criteria for the front-line use of alectinib being based on this regulatory limitation, the experts agreed that there is also a perception of wider knowledge and experience with this molecule as compared with brigatinib in clinical practice across the country, although the safety profile of the two compounds is considered similar.

With regard to survival outcomes, despite not yet being reached, results available confirm to be favorable for the use of alectinib [5].

The results available with alectinib in the active smoker subgroup (PFS HR 1.16, 95% CI 0.35–3.90, OS HR 1.97, 95% CI 0.38–10.20) are related to a very small group of patients in the ALEX study (n = 17) [5,13]. Hence, all faculty members agreed that no conclusion can be made based on these data.

As a last consideration, the experts pointed out that, with the future availability of front-line lorlatinib in Italy, a cost–opportunity evaluation could be helpful for pharmacoeconomic issues.

### 4.2. Patients’ Selection Criteria

Moving to treatment selection criteria, different clinical features were analyzed and discussed by the faculty as potential factors to guide front-line treatment choice: age, performance status, brain metastases, and other clinical considerations.

With regard to patients’ age, the experts agreed on the absence of specific treatment selection criteria exclusively based on age. However, in elderly patients, evaluation of treatment compliance, as well as the presence of a caregiver, are considered relevant for the treatment choice by all panel members (e.g., with regards to the daily number of pills to administer, this would not be in favor of alectinib for patients with treatment compliance deemed inadequate). In parallel, in the light of the future availability of lorlatinib, they agreed that the potential toxicities at the cognitive level could disadvantage lorlatinib, irrespective of age (to avoid further cognitive deterioration in the elderly, but also not to negatively affect the quality of life of young patients).

Similarly to age, no evidence supporting treatment selection based on performance status (PS) is available. However, in patients with poor PS, the experts considered it reasonable that the possibility of selecting a treatment based on a lower daily number of pills can favor the choice of brigatinib.

Brain metastases were then considered as a clinical factor to influence treatment choice. Panel members pointed out the absence of substantial differences in intracranial activity between brigatinib and alectinib (intracranial ORR 78% and 81%, respectively) [6,13]. Besides this, they are confident that lorlatinib, when available in Italy, will be the preferred treatment choice for patients with baseline brain metastases (intracranial ORR 83%; intracranial complete responses 72%) [20].

Other clinical factors were then reviewed to further evaluate treatment selection. In this view, the experts recommend considering a personalized treatment choice based on a specific comorbidity profile. As examples, they propose taking into account the risk for anemia with alectinib, as well as the risk for metabolic diseases with lorlatinib (to be balanced with respect to the frequently young age at NSCLC diagnosis). Furthermore, they recommend considering the pharmacological interactions in patients receiving polypharmacotherapy for comorbidities (e.g., drugs with central nervous system activity in patients with psychiatric disorders or direct-acting oral anticoagulants (DOACs) [22]).

### 4.3. The Treatment Sequence

Following the debate on front-line treatments, the discussion was moved to the evaluation of the therapeutic sequences applicable in Italy based on the available drugs and their local regulatory approvals.

Based on the previously mentioned assumption that no role is considered for the use of crizotinib or ceritinib, the panels did not propose any sequence starting with any of the two compounds. To the experts, the only sequences to be considered are the following:

- Alectinib, followed by lorlatinib, followed by platinum plus pemetrexed chemotherapy;
- Brigatinib followed by platinum plus pemetrexed chemotherapy;
- Lorlatinib—as soon as available—followed by platinum plus pemetrexed chemotherapy.

As previously discussed, in the light of literature data and regulatory approvals in Italy at the time of the webinar (July 2023), the first sequence is the preferred one according to all the faculty.

Of note, the experts gave a clear negative recommendation on the use of immune checkpoint inhibitors in combination with chemotherapy, based on the evidence indicating the absence of clinical and survival benefits across the available data [23].

### 4.4. Management of ALK TKI Treatment—Focus on Toxicity

With regard to the safety profiles of different ALK TKIs, two main comparisons were faced during the discussion: the first was based on the current treatment options in Italy of alectinib versus brigatinib; the second was instead related to the future treatment options of alectinib versus lorlatinib.

#### 4.4.1. Alectinib vs. Brigatinib

The main differences in the toxicity profiles of alectinib and brigatinib were identified as the risk of interstitial lung disease (ILD) in the first week with brigatinib [6], hepatotoxicity, and constipation with alectinib [13]. Furthermore, weight gain and anemia are mentioned by the experts as potential long-term toxicities with alectinib [13] that may have an impact on quality of life.

However, with respect to treatment selection, hepatotoxicity is not per se a limiting factor, although the panelists suggest evaluating it as an alert when considering alectinib in patients with chronic liver diseases. When considering hepatotoxicity, it is important to note that the isolated increase in bilirubine, in the absence of transaminases increase, is not an element of concern.

With regard to peripheral edema, a common adverse event with ALK TKIs, its adequate clinical management allows for no elements of concern in the treatment selection, according to the panel members.

Peculiar attention was then focused on ILD. Indeed, as there are alternative treatment options available, the experts recommend that the ILD risk with brigatinib should be considered, despite a rare event. In particular, in patients with severe respiratory comorbidities or those who received previous radiotherapy to the lungs and thorax, it is reasonable to avoid the choice of brigatinib.

In addition, they suggest that brigatinib should be avoided in cases of uncontrolled hypertension at baseline due to the rate of hypertension as a reported adverse event in the ALTA-1L trial (any grade hypertension: 17–27%, grade 3 or greater: 6–8%) [24].

#### 4.4.2. Alectinib vs. Lorlatinib

Peculiar toxicities of lorlatinib, in comparison with those already mentioned with alectinib, were identified as alterations in the lipidic profile, hypertension, and neurological/psychiatric alterations [19].

Considerations on hepatotoxicity and peripheral edema were the same as those made in the ‘alectinib vs. brigatinib’ section.

With respect to metabolic toxicity, the experts pointed out that, nowadays, there are no pieces of evidence of increased cardiovascular risk associated with lorlatinib-related metabolic alterations [20]. In their opinion, the management of alterations in the lipidic profile with statins and hypotriglyceride-lowering agents allows the safe use of lorlatinib. However, they commented that the metabolic risk could be a factor for treatment selection in patients with baseline severe cardiovascular comorbidities and/or uncontrolled hypertension.

Moving to neurological toxicity associated with lorlatinib, the expert opinion is that, given the availability of alternative treatment options, the presence of baseline neurological or psychiatric disorders (e.g., major depressive disorders, cognitive impairments, and neuropathies) can be taken into account for treatment choice. Conversely, in case of neurological disorders occurring de novo during treatment with lorlatinib, the experts agreed that dose reduction commonly allows for adequate control of such toxicity [20].

### 4.5. Improving the Therapeutic Process and Quality of Life according to Available Treatments in Italy

#### 4.5.1. Testing Strategies

Among the available testing strategies, the experts agreed that RNA-based next-generation sequencing (NGS) is the preferred method to identify *ALK* rearrangements in NSCLC [25]. However, they pointed out that the positive immunohistochemistry (IHC) expression of ALK protein, the testing method for inclusion criteria in the ALEX trial [13], is considered a valid and readily available diagnostic method to allow the prescription of ALK TKIs. With regards to fluorescence in situ hybridization (FISH), this is no longer considered a detection method for *ALK* rearrangements in the presence of reliable NGS results, but it still remains a confirmation technique in discordant IHC/NGS cases [26].

#### 4.5.2. Resistance Mechanisms

To the experts, the study of resistance mechanisms (either tissue or liquid biopsy) [27,28] is not helpful in clinical practice in the absence of treatment sequencing based on the results. However, they agreed that acquiring a new biopsy with molecular testing is a strategy to be considered in cases of early (primary) resistance to first-line ALK TKI, in order to provide better knowledge of the disease that would guide the subsequent treatment choice between second-line ALK TKI and chemotherapy (e.g., in the absence of confirmed *ALK* rearrangement or in the presence of co-occurring driver gene alterations [29]).

#### 4.5.3. Brain Evaluation

With regards to brain evaluation, based on the incidence of brain metastases in *ALK*-positive NSCLC, as well as knowing the CNS activity of ALK TKIs, all faculty members agreed that brain magnetic resonance imaging (MRI) with gadolinium is the recommended radiological exam for brain evaluation, both at baseline and at periodic assessments during oncological treatment [30,31].

#### 4.5.4. Baseline Brain Metastases Treatment

Following the imaging assessment, experts discussed the better approach to treat baseline brain metastases at NSCLC diagnosis in *ALK*-positive disease.

Their common consideration was to start ALK TKI alone in cases of asymptomatic brain metastases. Conversely, in the case of symptoms from CNS metastases, the indication for upfront radiation treatment, to be discussed with a radiation oncologist, should be based on the severity of the symptoms: in the case of controlled symptoms, the request for radiotherapy could be deferred at the time of an early brain radiological assessment following the start of ALK TKI treatment.

Indeed, despite most data being in favor of upfront ablative radiotherapy in patients with driver gene alterations [32,33], the risk of long-term radiation-related neurological toxicity or radionecrosis after brain irradiation should be considered in the light of long-term survival in patients with *ALK*-positive NSCLC [34].

Recently, an international survey was conducted among medical, clinical, and radiation oncologists, and neurosurgeons, regarding treatment recommendations for asymptomatic brain metastases in *EGFR* mutant or *ALK*-positive NSCLC patients, demonstrating a differential preferred approach according to different specialists in specific clinical scenarios [35]. Among 449 surveys, medical and clinical oncologists were more likely to recommend first-line TKI regardless of the number or size of asymptomatic brain metastases, compared with radiation oncologists and neurosurgeons. In patients with more than four brain metastases, the addition of radiotherapy to TKI was preferred among specialties [35].

#### 4.5.5. Oligoprogressive Disease

The last issue faced by the panelists was the clinical approach to oligoprogressive disease [36,37] during ALK TKI treatment. There was general agreement among all experts that the radiation treatment of the oligoprogressive sites, whenever feasible, is the best approach, continuing systemic ALK TKI beyond progression until clinical benefit is maintained.

## 5. Conclusions

Therapeutic options for *ALK*-rearranged advanced NSCLC has rapidly evolved in recent years. In Italy, the current scenario, based on local regulatory approvals, defines a role in the front-line setting for alectinib and brigatinib. Regulatory limitations and clinical expertise favor the choice of alectinib in most cases, thus allowing the subsequent use of lorlatinib. The future introduction of lorlatinib among the front-line treatment options will probably move the balancing of the use of the different compounds, at least in a proportion of patients. In addition, the recently announced positive results from the ALINA trial with alectinib in an adjuvant setting [38] are expected to introduce a new treatment scenario in the near future.

## Figures and Tables

**Table 1:** Approval scenario of ALK TKIs in the US, Europe, and Italy *.

	FDA	EMA	AIFA
**Crizotinib**	2011	2012 ^a^ 2015 ^b^	2015 ^a^ 2017 ^b^
**Ceritinib**	2014 ^c^ 2017 ^b^	2015 ^c^ 2017 ^b^	2017 ^c^ 2019 ^b^
**Alectinib**	2016 ^c^ 2017 ^b^	2017 ^c^ 2017 ^b^	2018 ^c^ 2018 ^b^
**Brigatinib**	2017 ^c^ 2020 ^b^	2018 ^c^ 2020 ^b^	2020 ^c^ 2020 ^b^
**Lorlatinib**	2018 ^d^ 2021 ^b^	2019 ^d^ 2021 ^b^	2021 ^d^

^a^ Previously treated with chemotherapy. ^b^ Front-line. ^c^ Post-crizotinib. ^d^ Post-ALK TKI. * Updated approvals by July 2023.

## Data Availability

The data presented in this study are available in this article.
